# Prognostic gene HLA‐DMA associated with cell cycle and immune infiltrates in LUAD

**DOI:** 10.1111/crj.13716

**Published:** 2023-11-16

**Authors:** Ya‐jie Huang, Jian‐kun He, Xiaoyang Duan, Ran Hou, Jian Shi

**Affiliations:** ^1^ Department of Medical Oncology The Fourth Hospital of Hebei Medical University Shijiazhuang Hebei China; ^2^ Department of Pathology The Fourth Hospital of Hebei Medical University Shijiazhuang Hebei China

**Keywords:** cell cycle, HLA‐DMA, immune infiltration, LUAD, TME

## Abstract

**Background:**

The dominant subclass of non‐small‐cell lung cancer (NSCLC) is lung adenocarcinoma (LUAD). The tumor microenvironment (TME) is a crucial feature of carcinogenesis and progression in LUAD. Furthermore, immune and stromal components of TME are crucial factors to investigating and curing LUAD. Thus, the study assessed the value of TME‐related genes for LUAD prognosis and immune infiltration.

**Methods:**

All data were downloaded from TCGA and GEO databases. The immune and stromal scores were downloaded from ESTIMATE, and the association between the scores and prognosis was explored by Kaplan–Meier survival analysis. Protein–protein interaction (PPI) network and univariate Cox regression were used to find TME‐related differentially expressed genes (DEGs), and HLA‐DMA was regarded as a prognostic hub gene. Western blot analyses, qRT‐PCR, and immunofluorescence were applied to verify HLA‐DMA expression in clinical samples. NSCLC cell lines were used to verify the effect of HLA‐DMA on cell proliferation and cell cycle distribution. At last, the alteration of immunotherapy response and TME transition caused by HLA‐DMA different expression were further studied.

**Results:**

The immune score was positively correlated with survival. The functional analyses suggested that TME‐related DEGs may be involved in the immune response. The expression level of HLA‐DMA was decreased in LUAD. In addition, HLA‐DMA expression was associated with several clinical features and was positively associated with survival. Furthermore, HLA‐DMA may suspend cell proliferation by regulating cell cycle. HLA‐DMA expression was closely associated with immune infiltration and positively correlated with TMB, indicating that patients with high HLA‐DMA level were more suitable for immunotherapy.

**Conclusion:**

These results reveal that HLA‐DMA might act as a biomarker for immune infiltration and immunotherapy response.

## Graphical abstract

HLA‐DMA is a TME monitor related to LUAD prognosis. HLA‐DMA may suspend cell proliferation by regulating cell cycle. Patients with high HLA‐DMA level are more sensitive for immunotherapy. HLA‐DMA is a biomarker for immune infiltration and immunotherapy response in LUAD.

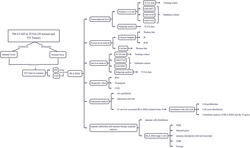

**Abbreviations**
AUC: area under the ROC curve
CCK‐8: Cell Counting Kit‐8
CI: confidence interval
DEG: differentially expressed gene
FDR: false discovery rate
GO: Gene Ontology
HLA‐DMA: major histocompatibility complex, class II, DM alpha
HR: hazard ratios
ICB: immune checkpoint blockade
KEGG: Kyoto Encyclopedia of Genes and Genomes
LUAD: lung adenocarcinoma
NSCLC: non‐small‐cell lung cancer
OS: overall survival
PPI: protein–protein interaction
RT‐qPCR: real‐time quantitative reverse transcription PCR
SNP: single nucleotide polymorphism
ssGSEA: single‐sample GSEA
TCGA: The Cancer Genome Atlas
TIDE: Tumor Immune Dysfunction and Exclusion
TIIC: tumor‐infiltrating immune cell
TME: tumor microenvironment
TMERG: TME‐related differentially expressed gene

## 1 BACKGROUND

According to the latest report by the National Cancer Center of China, lung cancer is the most common cancer, accounting for nearly 20.4% of new cancer cases.^1^ The prevalence of lung adenocarcinoma (LUAD) has rapidly increased recently.^2^ LUAD has become the chief cause of death among cancer patients, with more than 1 000 000 deaths per annum worldwide.^3^, ^4^ Although breakthrough advances in immunotherapies have achieved great success, limitations still exist in the improvement of non‐oncogene‐driven LUAD prognosis.^5^, ^6^, ^7^ Therefore, studies concentrating on the important therapeutic targets to assist essential clinical decision‐making are urgently needed.

The tumor microenvironment (TME) represents the cellular environment of tumors and consists of the extracellular matrix, immune cells, and stromal cells. The TME could predict immune checkpoint blockade (ICB) response and may be a potential therapeutic target in the future.^8^ Furthermore, studies have suggested that the immune cell infiltration in the TME was closely related to LUAD progression and immunotherapeutic response in LUAD.^9^, ^10^, ^11^, ^12^ In general, more efforts should be exerted to investigate the molecular characteristics of TME‐related prognostic genes to help improve LUAD outcome.

The current study found common differentially expressed genes (DEGs) filtered by immune and stromal scores; then we revealed that major histocompatibility complex, class II, DM alpha (HLA‐DMA) might serve as a TME‐related prognostic hub gene. HLA‐DMA is a protein‐coding gene that was recently reported as a diagnostic and prognostic cancer biomarker.^13^ In breast cancer, the upregulation of HLA‐DMA may lead to the activation and cytokine production of Th1 CD4^+^ T cells, eventually improving antitumor immunity and patient survival.^14^ However, the value of HLA‐DMA in LUAD has not been comprehensively evaluated from a variety of aspects. To better verify the effect of HLA‐DMA in LUAD, this study rigorously verified the prognostic value of HLA‐DMA in LUAD. And cell proliferation detection and cell cycle analyses verified that HLA‐DMA could suppress cell proliferation. In addition, the correlation between HLA‐DMA and tumor‐infiltrating immune cells (TIICs) were further investigated. Furthermore, the HLA‐DMA‐related immunotherapy response was valued. This research is the first to indicate that HLA‐DMA is a novel cell cycle‐ and TME‐related prognostic biomarker of immune infiltration in LUAD.

## 2 METHODS

### 2.1 Clinical specimens and data processing

The studies involving human participants and the experiments including any relevant details were admitted by the Ethics Committee of the Fourth Hospital of Hebei Medical University (No. 2019MEC100). All methods were confirmed to the Code of Ethics of the Declaration of Helsinki. The written informed consent to participate was obtained from participants. In total, 29 pairs of LUAD and matched adjacent normal lung tissues obtained from the Fourth Hospital of Hebei Medical University (Shijiazhuang, China) during December 2021 to July 2022 were frozen in liquid nitrogen until further analysis. The clinicopathological information was listed in Table S1.

First, publicly available RNA sequencing data (FPKM values) of LUAD were obtained from TCGA (https://portal.gdc.cancer.gov/projects/TCGA-LUAD); level 3 gene expression profile data of 594 LUAD cases (59 normal samples and 535 LUAD samples) and corresponding clinical data were included. Second, we merged the data through Perl (mRNA_merge.pl). To normalize the FPKM values, the gene expression data were transformed to log2 (value + 1). To further validate the results, the related data were extracted from the GEO database (https://www.ncbi.nlm.nih.gov/geo/): GSE10072 (33 LUAD and 33 paired adjacent nontumor samples), GSE19188 (45 LUAD and 65 healthy lung samples), GSE33532 (36 LUAD and paired adjacent nontumor samples), GSE50081 (128 LUAD samples), and GSE37745 (106 LUAD samples).

### 2.2 Immune and stromal score generation

The immune and stromal scores of LUAD were acquired from ESTIMATE (https://bioinformatics.mdanderson.org/estimate/), and the samples were grouped into low‐ and high‐score groups referring to the stromal and immune median scores.

### 2.3 Survival analysis

The relationship between the stromal and immune scores and overall survival (OS) rate was revealed by Kaplan–Meier survival analysis using the “survival” package in R software.

### 2.4 Score comparison among different clinical statuses

Wilcoxon rank sum or Kruskal–Wallis rank sum tests were used depending on the count of clinical status obtained from TCGA for the comparison.

### 2.5 TMERG identification

The LUAD samples were grouped into high score or low score according to the median of immune score and stromal score, respectively. The package “limma” in R software was utilized to conduct the difference analysis of the between gene expression, and TMERGs were excavated by the comparison between the high‐score and the low‐score samples.^15^ A false discovery rate (FDR) < 0.05 and |log fold change (logFC)| > 2 were defined as the selection criteria. The “VennDiagram” package was utilized to find common DEGs.

### 2.6 Functional analysis

Gene Ontology (GO; http://www.geneontology.org/) functional and Kyoto Encyclopedia of Genes and Genomes (KEGG; http://www.kegg.jp/kegg/pathway.html) pathway analyses were performed to understand the functions of the common up‐ and downregulated DEGs in LUAD through the “clusterProfiler” R package.^16^, ^17^, ^18^, ^19^

### 2.7 Protein–protein interaction (PPI) network and TME‐related prognostic hub gene identification

To obtain the interactions between the common up‐ and downregulated DEGs, a PPI network was constructed through STRING (https://string-db.org/)^20^ and rebuilt through Cytoscape software (version 3.7.2). The minimum required interaction score was set to 0.950. The Molecular Complex Detection plugin of Cytoscape was applied to screen out the significant modules from the PPI network (degree cutoff = 2, node score cutoff = 0.2, k‐core = 2, and maximum depth = 100). Furthermore, genes with node degrees ≥10 were defined as hub genes. The “survival” package in R software was used to conduct a univariate Cox regression, and the top 30 TME‐related prognostic DEGs were determined by the *P* value from small to large. The hazard ratios (HRs) and 95% confidence intervals (CIs) were generated by a Cox analysis. Venn diagrams were generated to find the intersection between PPI hub genes and TME‐related prognostic DEGs.

### 2.8 TME‐related prognostic hub gene analysis

The differential gene expression between the LUAD and control samples was evaluated by a Wilcoxon test. Furthermore, the difference in paired gene expression data between the LUAD and normal samples was analyzed by a Wilcoxon test. The association between gene expression and the clinical status of LUAD was analyzed by a Tukey honestly significant difference (HSD) test, Dunn's test, or Wilcoxon rank sum test according to the clinical status. The association between prognostic hub gene expression and OS was calculated through the packages “survminer” and “survival” in R software based on TCGA and GEO data. The survival rate of LUAD patients with different clinical features were generated using the “survminer” and “survival” packages in R software. Univariate and multivariate Cox analyses were used to determine the potential prognostic factors.

### 2.9 Diagnostic value of HLA‐DMA

The time ROC analysis was used to compare the predictive accuracy of HLA‐DMA. Multivariate and univariate cox were performed to develop the nomogram. A nomogram was constructed based on the results of multivariate Cox proportional hazards analysis to predict the 1‐, 3‐, and 5‐year overall recurrence through “rms” R package.

### 2.10 Survival‐associated HLA‐DMA‐related gene screening and functional analysis

A Spearman's correlation analysis was used to determine the HLA‐DMA‐related genes by R software. Then, the top 25 positively and 25 negatively HLA‐DMA‐related genes were ordered by the Spearman's correlation coefficient, and a heatmap of the top 50 HLA‐DMA‐related genes was generated by the package “ggplot2” in R. The “VennDiagram” package was utilized to screen the intersection genes of the top 500 HLA‐DMA‐related genes ordered by the Spearman's correlation coefficient and top 500 survival‐associated genes ordered by HR. Next, the intersection genes were subjected to GO and KEGG pathway analyses through the “clusterProfiler” R package. A PPI network of the intersection genes was constructed through STRING and rebuilt by the “igraph” R package. An intersection gene co‐expression matrix was produced by the “ggplot2” R package. The relationships between HLA‐DMA and the intersecting genes were calculated through a Spearman's correlation test.

### 2.11 HLA‐DMA‐related TIICs

Single‐sample GSEA (ssGSEA) was performed by using the GSVA package to obtain the fraction of TIICs. Then, all samples were divided into high‐ and low‐expression groups according to the median expression of HLA‐DMA. A Mann–Whitney *U* test was conducted to compare the difference in TIIC infiltration between the high‐ and low‐HLA‐DMA expression groups. The relationship between HLA‐DMA expression and TIIC infiltration was calculated by a Spearman's correlation analysis.

### 2.12 Significantly mutated genes and immunotherapy response based on HLA‐DMA expression

The TMB was calculated by tmb function of “maftools” package in R. SIGLEC15, TIGIT, CD274, HAVCR2, PDCD1, CTLA4, LAG3, and PDCD1LG2 were selected to be immune checkpoint‐related transcripts, and the expression values of the eight genes were got from the TCGA‐LUAD data. The statistical difference of the eight genes in HLA‐DMA high/low and normal groups was compared through the Kruskal–Wallis test. Potential ICB response was forecast by Tumor Immune Dysfunction and Exclusion (TIDE) algorithm. The data of mutations were downloaded and visualized using the “maftools” package. Genes with higher mutational frequency detected of LUAD patient in histogram were shown.

### 2.13 Cell culture

The A549 and H1975 human LUAD cell lines and BEAS‐2B human normal lung fibroblast cell lines used in this study were purchased from the Cell Bank of Type Culture Collection of the Chinese Academy of Science (Shanghai Institute of Cell Biology, China) and authenticated by STR typing. The A549 were cultured in Ham's F‐12K medium with 10% fetal bovine serum (FBS, Gibco, Thermo Fisher Scientific, Waltham, MA). H1975 were cultured in RPMI 1640 medium (Gibco, Life Technologies, California, USA) with 10% fetal bovine serum. The BEAS‐2B were cultured in were cultured in RPMI 160 medium (Invitrogen, Shanghai, China) supplemented with 10% FBS. The cells were placed in a CO_2_ incubator (SANYO Electric Co., Ltd., Japan) with constant 90% humidity and 5% CO_2_.

### 2.14 Cell transfection

The full‐length HLA‐DMA cDNA sequence was synthesized and cloned into pcDNA3.1 plasmid vector (NewHelix, China). The pcDNA3.1‐HLA‐DMA vector or pcDNA3.1 plasmid vector was transfected into A549 and H1975 cell lines with Lipo8000™ transfection Reagent (Beyotime, China), and the cells that stably expressed HLA‐DMA were identified by a Western blot analysis.

### 2.15 Cell proliferation assay

Cell proliferation was examined with a Cell Counting Kit‐8 (CCK‐8, Beyotime, China) assay according to the manufacturer's protocols. A549 and H1975 cells were transfected with the corresponding vector, seeded into 96‐well plates and cultured for 24, 48, and 72 h. Next, CCK‐8 reagent (approximately 10 μL) was added to each well, and the cells were incubated for an additional 1 or 2 h. This experiment was performed in triplicate.

### 2.16 Cell cycle analysis

Cells were collected and fixed in 70% ethanol at 4°C overnight. Then, the cells were treated with RNase A (50 μg/mL, Fuyuanbio, China) and stained with DAPI Staining Solution (DAPI, 10 μg/mL, Beyotime, China) for 30 min at 37°C after washing with PBS. The distribution of the cell cycle phases was calculated by a FACSCalibur flow cytometer (Becton, Dickinson and Company, CA). The phase ratio (%) was regarded as the percentage of cells in the G1/S/G2 phase. Each experiment was conducted in triplicate.

### 2.17 Real‐time quantitative reverse transcription PCR (RT‐qPCR)

The total RNA was extracted from 15 paired NSCLC tissues using TRIzol reagent (Invitrogen, Carlsbad, California, USA). The total RNA was then transcribed to cDNA using Servicebio RT First Strand cDNA Synthesis Kit (G3330; Wuhan, China) according to the manufacturer's instructions. The reverse transcription procedure was: 5 min at 25 and 42°C for 30 min and then 5 s at 85°C. Real‐time quantitative reverse transcription PCR (RT‐qPCR) was performed on a CFX96 Touch Real‐Time PCR Detection System (Bio‐Rad, China). The RT‐qPCR system contained 7.5 μL 2 × SYBR Green qPCR Master Mix, 0.75 μL forward primer, 0.75 μL reverse primer, 2 μL cDNA product, and 4.0 μL water nuclease‐free. The reaction protocol was 95°C for 30 s, followed by 40 cycles at 95°C for 15 s and 60°C for 30 s. The primers were synthesized by Servicebio (Wuhan, China). The 2^−ΔCq^ method was used to demonstrate the expression levels of HLA‐DMA. All experiments were conducted in triplicate. The primer sequences used for RT‐qPCR were GAPDH: 5′‐GGAAGCTTGTCATCAATGGAAATC‐3′,5′‐TGATGACCCTTTTGGCTCCC‐3′; HLA‐DMA: 5′‐TGTCCAGAGGGTTTCCTATCG‐3′,5′‐CTGCCAGTTCACTGTCAGCAT‐3′.

### 2.18 Western blotting

Five LUAD samples with paired adjacent normal lung tissue samples and LUAD cells were lysed with a protease inhibitor, phosphatase inhibitor, and RIPA cleavage buffer (Thermo Fisher Scientific, Shanghai, China). The protein lysates were separated by sodium dodecyl sulfate–polyacrylamide gel electrophoresis (SDS‐PAGE) (Servicebio, Wuhan, China) and imprinted on polyvinylidene fluoride (PVDF) membranes (Millipore, Billerica, MA, USA) for analysis. The samples were incubated with anti‐HLA‐DMA (1:1000 dilution, #PA5‐22365; Thermo Fisher, Shanghai, China) and anti‐GAPDH (1:2000 dilution, GB15002; Servicebio, Wuhan, China) antibodies overnight at 4°C. Horseradish peroxidase (HRP)‐conjugated secondary antibodies (1:5000 dilution; GB25301, Servicebio, Wuhan, China) were added for 2 h at room temperature. Protein bands were visualized using ECL Plus Western Blotting Substrate under chemiluminescence system (Tanon 5200). The analysis of Western blot result was conducted by Image J software.

### 2.19 Immunofluorescence

Nine paraffin‐embedded LUAD and paracarcinoma tissues were used for immunofluorescence (IF). Each tissue was incubated overnight with a primary antibody against HLA‐DMA (1:100 dilution, #PA5‐22365; Thermo Fisher, Shanghai, China) at 4°C. After washing with PBS, coverslips were treated with Cy3 conjugated Goat Anti‐rabbit IgG (1:300 dilution, GB21303, Servicebio, Wuhan, China) and DAPI staining. The images were photographed under a Nikon Eclipse C1 Ortho‐Fluorescent Microscopy (Nikon Instruments Inc., Japan). The results were analyzed and scored by three pathologists who were blinded to the classification of the samples. ImageJ software was used to analyze the intensity of staining using a semiquantitative integration method.

### 2.20 Statistical analysis

All data were exhibited as mean ± standard deviation (SD). The results of HLA‐DMA in LUAD patients and paracarcinoma tissues in Western blot analysis were detected by one‐way ANOVA to test difference between LUAD and LUAD groups or different cell lines. The difference of HLA‐DMA expression level in IF between NSCLC and paired normal tissue was determined by paired‐samples Test. The difference of HLA‐DMA mRNA expression level between NSCLC and paired normal tissue was determined by Wilcoxon signed rank test. The correlation between HLA‐DMA and S phase‐related genes was conducted by Spearman correlation analysis. Statistical significance was considered when *P* < 0.05.

## 3 RESULTS

### 3.1 Stromal and immune scores of the study population and correlation with clinical features

Workflow of the study is presented in Figure S1. The stromal and immune scores were ranged from −1959.31 to 2098.77 and −1355.85 to 2905.3. The immune scores of the LUAD patients were positively correlated with the OS rate (log‐rank *P* = 0.01 in Figure S2A). However, the correlation between the stromal scores and OS rate showed no significant differences at the level of *P* < 0.05 (Figure S2B).

The immune score was negatively correlated with the T and M classification (*P* = 0.005 in Figure S2C and *P* = 0.035 in Figure S2E), but there were no significant differences between the immune score and N classification (*P* = 0.433 in Figure S2D). Higher immune scores were markedly correlated with earlier stages (*P* = 0.034 in Figure S2F). The correlation between the stromal scores and T, N or stages classification indicated no significant differences at the level of *P* < 0.05 in Figure S2G,H,J, respectively. The stromal score only showed a negative correlation with the M classification (*P* = 0.014 in Figure S2I). Overall, the fraction of TME components were connected with the metastasis and tumor size of LUAD.

### 3.2 TME‐related DEG screening and functional enrichment analysis

According to the median of stromal score, the samples were divided into high/low‐score group, 2192 upregulated genes and 2680 downregulated genes were found (Figure S3A). Similarly, 1589 upregulated genes and 161 downregulated genes of immune score were obtained (Figure S3B). The Venn diagrams indicate that 620 DEGs were commonly upregulated, and 35 DEGs were commonly downregulated (Figure S3C,D).

The KEGG functional analysis indicated that these DEGs were involved in cytokine–cytokine receptor interactions (Figure S3E). The GO functional analysis verified that these DEGs were enriched in the immune system process and immune response, indicating that the DEGs were closely associated with the LUAD immune response (Figure S3F–H).

### 3.3 HLA‐DMA as a TME‐related prognostic hub gene and gene expression validation

The PPI network consisted of 472 nodes and 416 edges. Then, the network was constructed by Cytoscape, and the top 3 significant modules are displayed in Figure S4A. Among the 472 nodes, 21 central node genes were regarded as hub genes based on the criterion of a filtering degree ≥10 (Figure S4B). The top 30 factors ranked by the *P* value were showed in Figure S4C. Finally, an intersection analysis between the 21 central node genes and the top 30 factors was performed, and the common gene HLA‐DMA was ultimately selected as the prognostic hub gene (Figure S4D).

HLA‐DMA in the normal samples was obviously higher than that in the LUAD group (log‐rank *P* = 2.6e‐08; Figure S5A). In addition, paired HLA‐DMA expression in LUAD was lower than that in the normal controls (*P* = 5.8e‐07; Figure S5B). Furthermore, HLA‐DMA expression was significantly related to the T stage (*P* = 1.3e‐04; Figure S5C), initial treatment outcome (*P* = 0.01, Figure S5D), OS event (P = 2e‐03, Figure S5E), age (P = 4e‐04, Figure S5F) and smoking (*P* = 4.3e‐06; Figure S5G). Data in GSE10072, GSE19188, and GSE33532 proved that HLA‐DMA level in LUAD was higher in normal control (*P* < 0.05; Figure S5H–J). The comparison between LUAD and control demonstrated that HLA‐DMA expression was lower in LUAD (*P* < 0.05; Figure S5K–M).

The HLA‐DMA expression in tumors was obviously lower than that in matched adjacent normal lung tissues in Western blot results (*P* < 0.001; Figure S6A). The qRT‐PCR indicated lower HLA‐DMA expression in LUAD (*P* = 6.1e‐05; Figure S6B). In addition, the IF also exhibited that the HLA‐DMA was downregulated in LUAD (*P* < 0.001; S6C,D). Besides, the expression of HLA‐DMA was obviously higher in BEAS‐2B cells compared with that in A549 and H1975 cells (*P* < 0.001; Figure S6E). The above findings verified that the HLA‐DMA was decreased in LUAD.

### 3.4 Higher HLA‐DMA expression indicates longer OS in LUAD

As shown in Figure S7A, higher HLA‐DMA expression was related to a longer OS in the LUAD patients (HR = 0.61, *P* = 0.001). Then, the subgroup analysis shown in Figure S7B–E indicated that a higher expression of HLA‐DMA was significantly related to a better prognosis in patients with the following clinical statuses: age over 65 years (*P* = 0.019), T2 (*P* < 0.001), Stage I (*P* = 0.007) and smoking (*P* = 0.005). And patients in higher HLA‐DMA group had a longer OS (P < 0.05, Figure S7F–G) in the validation cohort. The univariate Cox analysis showed the high HLA‐DMA level was obviously correlated with a longer OS (HR = 0.599, 95% CI = 0.446–0.804) (Table 1). These outcomes suggest that higher HLA‐DMA patients had a longer OS.

**TABLE 1:** Univariate and multivariate Cox regression analyses of the clinical characteristics associated with OS in LUAD in TCGA.

Characteristics	Total(N)	Univariate analysis		Multivariate analysis	
		Hazard ratio (95% CI)	P value	Hazard ratio (95% CI)	P value
HLA‐DMA	504				
Low	254	Reference			
High	250	0.599 (0.446–0.804)	<0.001	0.700 (0.465–1.053)	0.087
Pathologic stage	496				
Stage I and stage II	389	Reference			
Stage III and stage IV	107	2.624 (1.926–3.576)	<0.001	1.562 (0.926–2.634)	0.095
T stage	501				
T1	168	Reference			
T3 and T2 and T4	333	1.668 (1.184–2.349)	0.003	1.500 (0.921–2.442)	0.103
N stage	492				
N0	325	Reference			
N1 and N2 and N3	167	2.606 (1.939–3.503)	<0.001	1.690 (1.082–2.641)	0.021
M stage	360				
M0	335	Reference			
M1	25	2.111 (1.232–3.616)	0.007	1.102 (0.481–2.527)	0.818
Primary therapy outcome	419				
PR and CR	314	Reference			
PD and SD	105	2.786 (1.978–3.924)	<0.001	2.845 (1.871–4.326)	<0.001
Gender	504				
Female	270	Reference			
Male	234	1.060 (0.792–1.418)	0.694		
Smoker	490				
No	71	Reference			
Yes	419	0.887 (0.587–1.339)	0.568		
Age	494				
< = 65	238	Reference			
>65	256	1.228 (0.915–1.649)	0.171		

### 3.5 Diagnostic value of HLA‐DMA

The area under the 1‐year ROC curve (AUC) was 0.657, as shown in Figure S8A. Cox regression demonstrated that HLA‐DMA was an independent risk factor (Figure S8B,C). A nomogram containing HLA‐DMA expression and clinical features was constructed to predict the 1‐, 3‐, and 5‐year survival probability (Figure S8D). The calibration analysis showed that the curves at 1‐, 3‐ and 5‐year were close to the ideal line and verified the accuracy of the nomogram (Figure S8E).

### 3.6 Functional analysis of survival‐associated HLA‐DMA‐related genes

**FIGURE 1:**
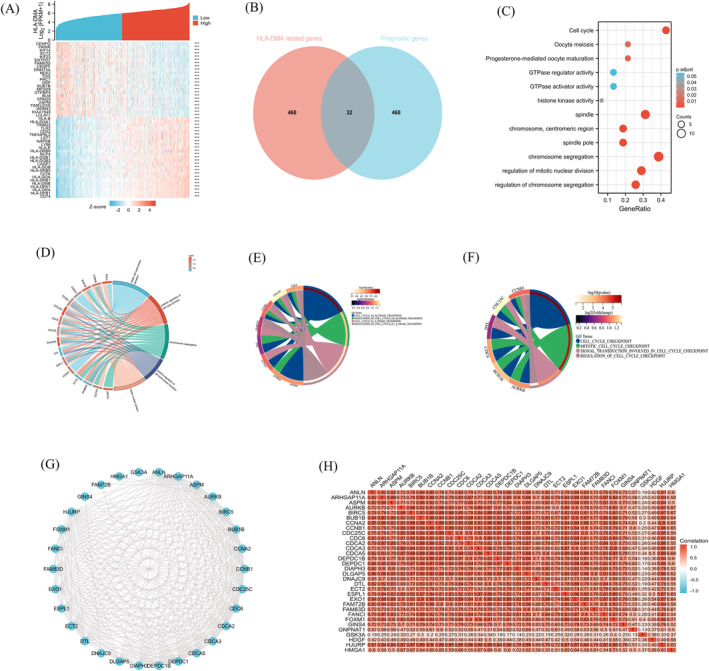
HLA‐DMA functional and interaction network analysis of HLA‐DMA‐related genes. (A) Heatmap showed the top 50 genes of LUAD that were positively and negatively related to HLA‐DMA. Red represents positively related genes and blue represents negatively related genes. (B) Venn diagram of the HLA‐DMA‐related genes and the survival‐related genes in LUAD. (C) GO and KEGG pathway analyses of the HLA‐DMA‐related prognostic genes in LUAD. (D) The biological processes of cell cycle involved by HLA‐DMA‐related genes. (E) The biological processes of cell cycle phase transition involved by HLA‐DMA‐related genes. (F) The biological processes of cell cycle checkpoint involved by HLA‐DMA‐related genes. (G) HLA‐DMA‐related prognostic gene interaction network. (H) HLA‐DMA‐related prognostic gene co‐expression matrix.

**FIGURE 2:**
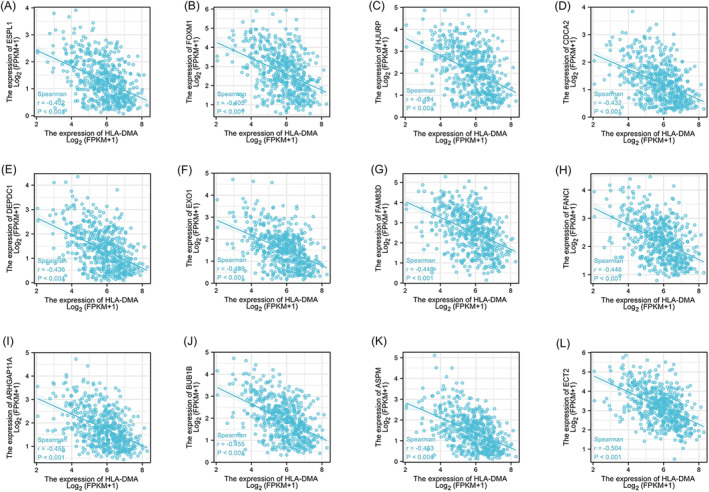
The correlation analysis between HLA‐DMA and the cell cycle related genes in LUAD. (A) ESPL1, (B) FOXM1, (C) HJURP, (D) CDCA2, (E) DEPDC1, (F) EXO1, (G) FAM83D, (H) FANCI, (I) ARHGAP11A, (J) BUB1B, (K) ASPM, (L) ECT2.

A heatmap was constructed to show the top 50 genes positively (red) and negatively (blue) related to HLA‐DMA (Figure 1A). A Venn diagram screened 32 survival‐associated HLA‐DMA‐related genes (Figure 1B), and functional analyses of these 32 genes revealed that cell cycle signaling and spindle and chromosome segregation were enriched (Figure 1C). In addition, further analysis of the cell cycle‐related functions revealed that 16 HLA‐DMA‐related genes were enriched in positive regulation of cell cycle process (Figure 1D). Figure 1E further demonstrated the eight HLA‐DMA‐related genes were involved in G1/S phase and G2/M phase transition, and Figure 1F showed that six HLA‐DMA‐related genes were enriched in the cell cycle checkpoint. The PPI network showed a stronger enrichment network that was closely related to cell cycle (Figure 1G). The gene coexpression correlation analysis demonstrated that the proteins had a significant positive correlation with each other (Figure 1H). The relationship between HLA‐DMA and cell cycle‐related genes was calculated by a Spearman's correlation test. The results showed that 12 genes among the cell cycle‐related genes were negatively related to HLA‐DMA expression (*r* < −0.4, *P* < 0.001; Figure 2A–L), indicating that HLA‐DMA might participate in the regulation of the cell cycle.

### 3.7 HLA‐DMA may inhibit NSCLC cell proliferation by inducing S phase arrest

**FIGURE 3:**
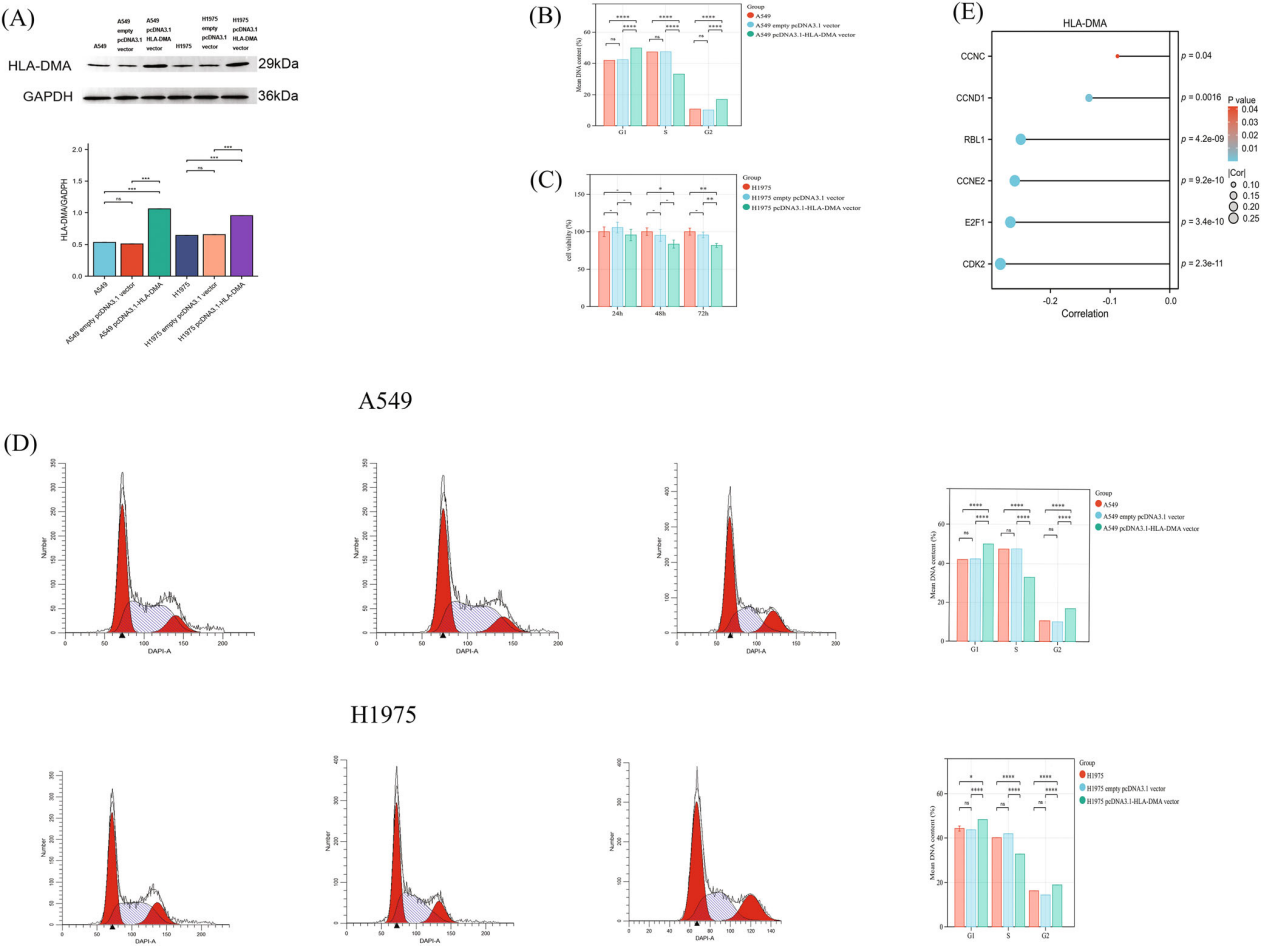
HLA‐DMA affected the LUAD cell proliferation by regulating cell cycle. (A)Western blot presented the A549 cells and H1975 cells over‐expressed HLA‐DMA after cell transfection. CCK‐8 was used to detect the proliferation ability of A549 cells (B) and H1975 cells (C), and flow cytometry was used to detect the cell cycle distribution of A549 and H1975 cells (D). (E) Correlation between HLA‐DMA and the S phase‐related genes. *P < 0.05; **P < 0.01; ***P < 0.001; ****P < 0.0001; ns indicated *P* > 0.05.

The expression of HLA‐DMA was increased after the transfection with the pcDNA3.1‐HLA‐DMA compared with empty pcDNA3.1 vector or control in H1975 and A549 cells (*P* < 0.001; Figure 3A). The increased expression of HLA‐DMA led to an obvious reduction in the viability of H1975 and A549 cells at 48 and 72 h time point according to the CCK‐8 assay (*P* < 0.05; Figure 3B,C).

Flow cytometry was used to examine the changes in cell cycle induced in H1975 and A549 cells. The percentage of cells in S phase decreased from 47.5 to 33 in empty vector transfected A549 cells and pcDNA3.1‐HLA‐DMA vector transfected A549 cells. In the meanwhile, the percentage of cells in S phase decreased from 41.9 to 32.7 in empty vector transfected H1975 cells and pcDNA3.1‐HLA‐DMA vector transfected H1975 cells. The upregulation of HLA‐DMA markedly decreasing the ratio of cells in the S phase cells (*P* < 0.001; Figure 3D), resulting in cell cycle arrest compared with the control. The specific functions of S phase‐related markers were provided in Table S2. Furthermore, the correlation between HLA‐DMA and S phase‐related markers was shown in Figure 3E, higher HLA‐DMA expression accompanied lower level of CCNC/CCND1/RBL1/CCNE2/CDK2/E2F1(*P* < 0.05; Figure 3E). These results indicated that HLA‐DMA may inhibit cell proliferation by arresting cell cycle at S phase.

### 3.8 HLA‐DMA‐related TIICs and ICB response prediction

**FIGURE 4:**
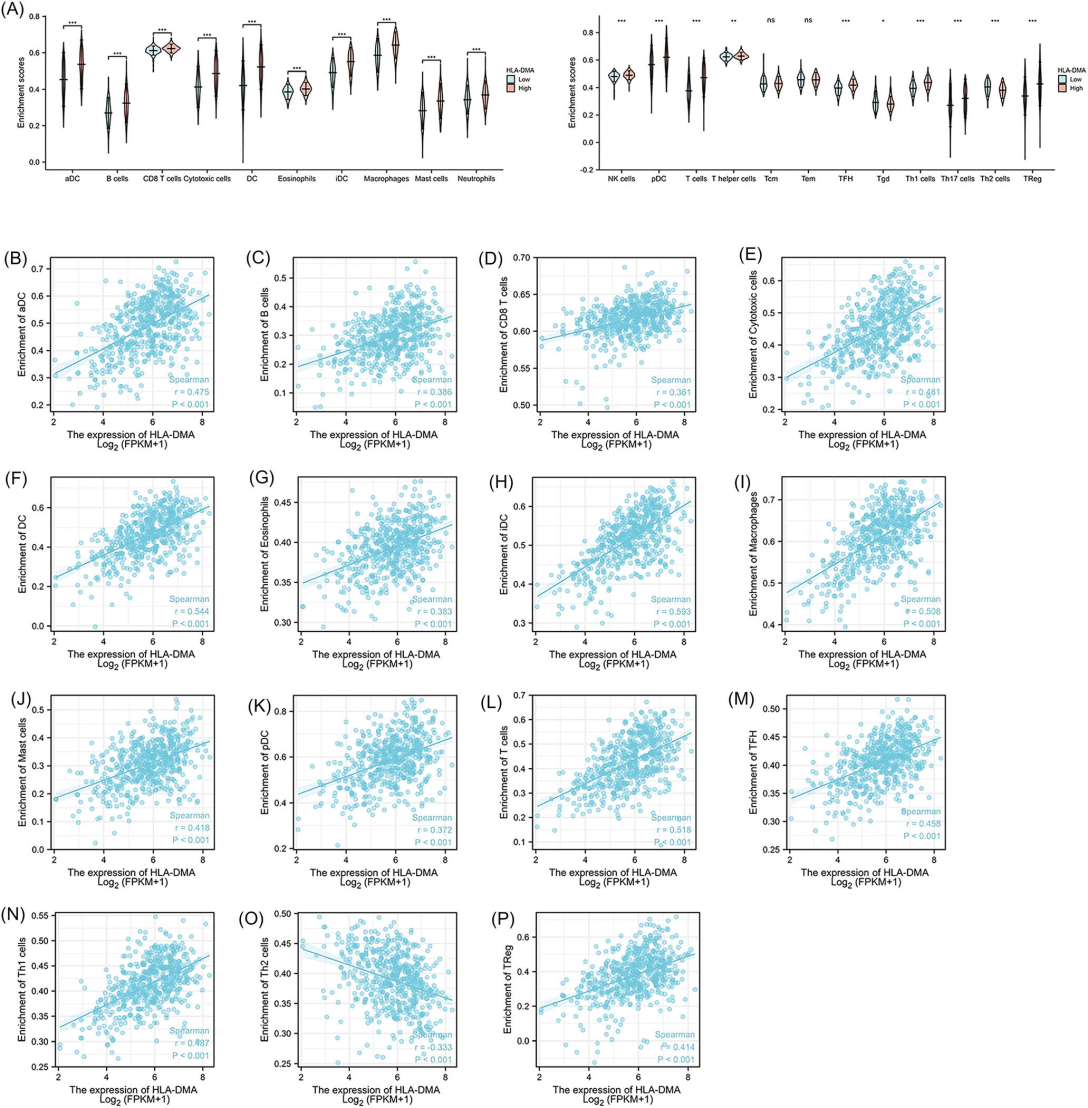
Correlation analysis of HLA‐DMA expression and immune infiltration LUAD. (A) Differential distribution of immune cells in patients with high‐HLA‐DMA expression and low‐HLA‐DMA expression. (B–P) Correlation between the HLA‐DMA expression level and immune cell infiltration in LUAD: (B) aDC cells, (C) B cells, (D) CD8 T cells, (E) cytotoxic cells, (F) DC, (G) eosinophils, (H) iDC cells, (I) macrophages, (J) mast cells, (K) pDC, (L) T cells, (M) TFH, (N) Th1 cells, (O) Th2 cells, (P) Treg. *P < 0.05; **P < 0.01; ***P < 0.001, ns, no significance.

A Wilcoxon rank sum test was performed to compare the immune infiltration difference between the two groups (high vs. low), and the results are shown in Figure 4A. Compared with the HLA‐DMA high group, the infiltration levels of aDCs, B cells, CD8 T cells, cytotoxic cells, DCs, eosinophils, iDCs, macrophages, mast cells, neutrophils, NK cells, pDCs, T cells, T helper cells, TFH cells, Th1 cells, Th17 cells, and Tregs were obviously lower in the low group (*P* < 0.01). In contrast, Th2 cell and Tgd infiltration were higher in the low group (*P* < 0.05). The Spearman's correlation analysis revealed a correlation between HLA‐DMA expression and the immune infiltration level (Figure 4B–P), and Th2 cells were negatively associated with the HLA‐DMA expression levels (*r* < −0.3, *P* < 0.001). In contrast, 14 types of TIICs were positively correlated with the HLA‐DMA expression levels, including aDCs, B cells, CD8 T cells, cytotoxic cells, DCs, iDCs, macrophages, mast cells, neutrophils, pDCs, T cells, TFH cells, Th1 cells, and Tregs (*r* > 0.3, *P* < 0.001).

**FIGURE 5:**
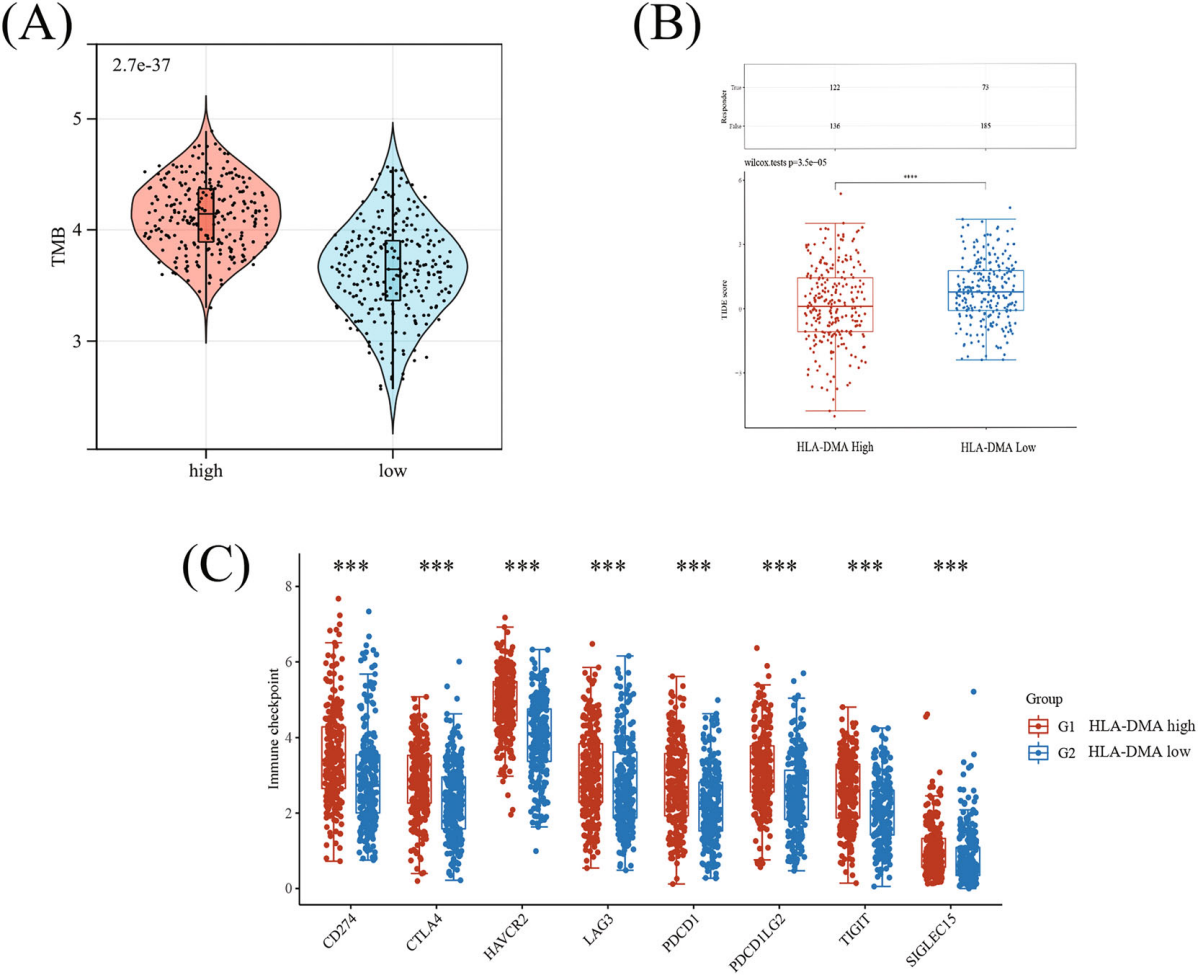
Comparison of TMB, TIDE and immune checkpoint‐relevant genes expression between high‐ and low‐HLA‐DMA expression patients. The differences of the TMB (A), TIDE score (B), and immune checkpoint‐relevant gene expression (C) in high‐ and low‐HLA‐DMA expression patients.

The high HLA‐DMA expression patients were accompanied with higher TMB (Figure 5A; *P* < 0.001). In Figure 5B, high HLA‐DMA group with lower TIDE score, indicating better ICB response and longer survival time. The HLA‐DMA high group accompanied with higher immune checkpoint‐relevant genes expression (Figure 5C, P < 0.001), which suggested that high HLA‐DMA group may have better immunotherapy response.

### 3.9 Genomic alterations in high‐ and low‐ HLA‐DMA patients

**FIGURE 6:**
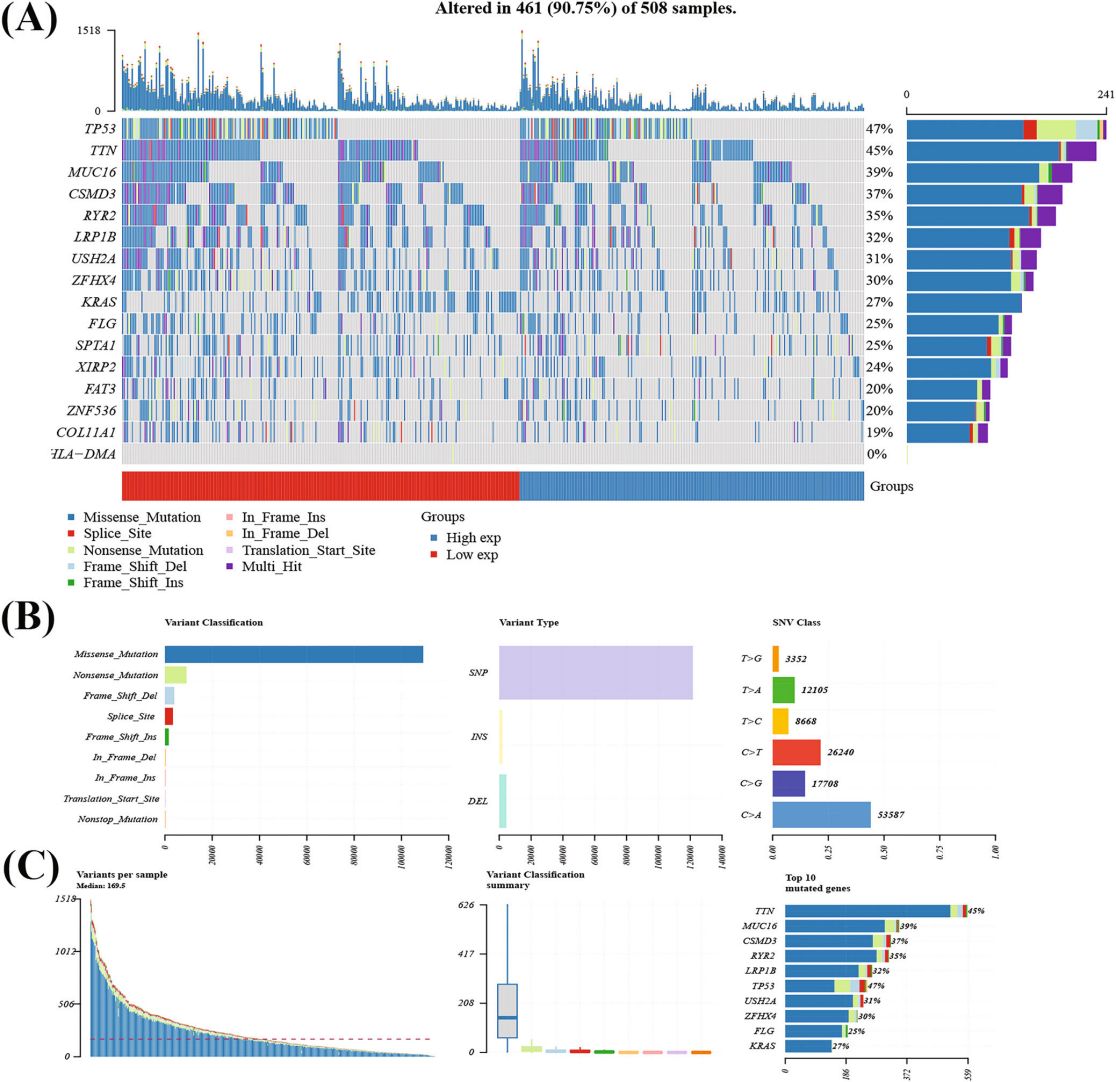
Evaluation of genomic alterations. (A) Waterfall plot shows mutation information for each gene for each sample. Color annotation of various cancer types are shown at the bottom. The barplot above the legend shows the number of mutation burden. (B) Cohort summary plot shows the distribution of variants according to variant classification, type, and SNV class. (C) Bottom part (from left to right) indicates mutation load of each sample (variant classification type). Stacked bar graph shows the top 10 mutated genes.

The top 15 mutated genes in the high‐ and low‐HLA‐DMA expression (Figure 6A) patients were compared. The mutation frequencies of genes, including TTN(45%) and TP53(47%), were higher in the patients with low HLA‐DMA expression. Missense mutations were the top common type of mutation, single nucleotide polymorphism (SNP) was the fore, and the primary type of mutation was T > G (Figure 6B). In Figure 6C, the amount of mutations per sample was presented, and each kind of mutation was represented by a color in the box diagram.

## 4 DISCUSSION

Increasing numbers of potential targets for increasing the LUAD survival rate and predicting prognosis have emerged.^21^ The milieu of the TME contributes to therapeutic resistance, inflammation, immune suppression, and distant metastasis of lung cancer as previously reported.^22^ In addition, the diversity of the TME types in LUAD may lead to different OS rates.^23^, ^24^ However, few studies have investigated the significance of TME‐related genes in LUAD prognosis and ICB response. Thus, this study attempted to discover TME‐related genes that may significantly predict the prognosis and ICB response in LUAD using TCGA and GEO database data.

First, the results indicated that patients with higher immune score might have a longer OS. Then, GO and KEGG results presented that the TME‐related DEGs were primarily related to LUAD immune response. The subsequent analysis suggested that HLA‐DMA was a TME‐related prognostic hub gene. By collecting samples from LUAD patients, we verified that HLA‐DMA expression was decreased in LUAD tissues. Further analysis indicated that higher HLA‐DMA expression is correlated with a better OS in LUAD. Meanwhile, a low HLA‐DMA expression level is related to poor clinical features. Overall, HLA‐DMA may act as a potential biomarker for LUAD prognosis. To further investigate the potential mechanism by which HLA‐DMA affects LUAD prognosis, the 32 survival‐associated HLA‐DMA‐related genes were found to be enriched in the cell cycle through GO and KEGG analyses, furthermore, GO enrichment analysis indicated that G2/M phase, G1/S phase transition, and cell cycle checkpoints played an essential role in HLA‐DMA‐related genes. CCND1 and CCNE2 implicated in promoting G1/S transition,^25^, ^26^ our results showed the significant negative correlation between HLA‐DMA and CCND1/CCNE2, which also indicated HLA‐DMA may inhibit cell cycle process by causing G1/S arrest. The CCK‐8 and flow cytometry were conducted to explore the effect of HLA‐DMA on cell cycle, and the results revealed that HLA‐DMA overexpression suppressed the proliferation of LUAD cells by arresting cell cycle at the S phase. Previous research found that cyclin C (CCNC) gene mRNA reached a maximal level in the S phase, and contributed to the regulation of G1/S and G2/M phases of the cell cycle.^27^ RBL1 is an important cell cycle regulator that controls the G1/S transition by binding to E2F transcription factors and blocking the transactivation of genes required for S‐phase entry.^28^ In our study, HLA‐DMA was negatively correlated with CCNC/RBL1/E2F1, which also suggested that HLA‐DMA overexpression could restain cell cycle process at S phase. Combined with these results, HLA‐DMA may retard LUAD cell proliferation through the regulation of cell cycle.

More importantly, subsequent bioinformatics analysis verified that HLA‐DMA expression was significantly relevant to immune cell infiltration in LUAD, and HLA‐DMA expression was positively correlated with CD8+ T cells. CD8+ T cells were correlated with a positive prognosis,^29^ indicating that HLA‐DMA may shift LUAD prognosis and immunotherapy sensitivity by conditioning the TME status. Based on the previously reported results, high HLA‐DMA level patients with high CD8+ T cells infiltration may present a “hot” immune microenvironment,^30^ which could improve immunogenicity, bringing a relatively higher rate of immunotherapy response. Besides, the study also find the positive relationship between HLA‐DMA and macrophages, and macrophages play an important role in antitumor immunity and better clinical outcome prediction in lung cancer.^31^, ^32^ TMB could reflect the mutations of DNA in cancer cells, which is measured by mutations per megabase (mut/Mb),^33^ and TMB may serve as a guidance in the best immune checkpoint inhibitors selection.^34^ HLA‐DMA was positively related with TMB in our findings, suggesting that patients with high HLA‐DMA level may receive benefits of PD‐1/PD‐L1 inhibitors. Exploring the TME‐related genes of LUAD could facilitate the identification of patients who are sensitive to immunotherapy and increase the rate of ICB response.

However, further real‐world clinical studies are necessary to support the conclusions above. In general, relevant research should be performed to provide more comprehensive insight into the accuracy of the HLA‐DMA expression analysis, new treatments targeting HLA‐DMA, and the possible associations between the TME and LUAD prognosis.

## 5 CONCLUSIONS

HLA‐DMA was identified as a novel TME‐related prognostic hub gene, which was developed and applied as a TME monitor and transition for LUAD prognosis. HLA‐DMA participated in LUAD cell cycle regulation and suspended cell proliferation. HLA‐DMA expression was closely associated with immune infiltration and negatively correlated with TIDE scores, which might bring a new insight for ICB response precision.

## AUTHOR CONTRIBUTIONS

*Conceptualization*: Ya‐jie Huang and Jian Shi. *Methodology*: Jian‐kun He. *Software*: Ya‐jie Huang. *Validation*: Xiaoyang Duan, Ran Hou, and Jian Shi. *Investigation*: Ya‐jie Huang. *Resources*: Jian‐kun He. *Data curation*: Ya‐jie Huang. *Writing—original draft preparation*: Ya‐jie Huang. *Writing—review and editing*: Xiaoyang Duan. Visualization: Ran Hou. *Supervision*; Jian Shi. *Project administration*: Ya‐jie Huang. Funding acquisition: Jian Shi. All authors read and approved the final manuscript.

## CONFLICT OF INTEREST STATEMENT

The authors declare that they have no competing interests.

## ETHICS STATEMENT

Ethics approval (No. 2019MEC100) was granted by the Ethics Committee of Fourth Hospital of Hebei Medical University. All methods were performed in accordance with the Code of Ethics of the World Medical Association (Declaration of Helsinki). Written informed consent was provided by all patients.

## Supporting information


**Figure S1.** Analysis workflow of this study.
**Figure S2.** The correlation between scores and the survival/clinicopathological staging characteristics of LUAD patients. (A) Kaplan–Meier survival analysis of high‐ or low‐ score group determined by the median of Immune Score. Log‐rank P = 0.01. (B) Kaplan–Meier survival curve for Stromal Score. Log‐rank P = 0.064. (C‐F) The correlation of Immune Score with T, N, M classification and clinical stage. (G‐J) The correlation of Stromal Score with T, N, M classification and clinical stage.
**Figure S3.** Vocalno plot, Venn plots, GO and KEGG analysis for TME‐related DEGs. (A) Based on Stromal Score comparison, 2192 genes were up‐regulated and 2680 genes down‐regulated in the high score than the low score group after propensity analysis using limma package algorithm. (B) Vocalno plot for DEGs in Immune Score, similar with (A). (C‐D) Venn plots showing common down‐regulated and up‐regulated DEGs shared by Immune Score and Stromal Score. (E) KEGG enrichment analysis for 655 TME‐related DEGs. (F‐H) Functional enrichment analysis including GO: BP, GO: CC, GO: MF, respectively.
**Figure S4.** PPI network and univariate COX. (A) Top three modules in the PPI networks. (B) The 21 genes with node degrees≥10. (C) Univariate COX regression analysis with 655 DEGs, listing the top 30 significant factors with P value from small to large. (D) Venn plot intersected the TME‐related prognostic hub gene shared by leading 21 nodes in PPI and top 30 significant factors in COX.
**Figure S5.** The HLA‐DMA expression in LUAD and control in TCGA and validation in GEO database. (A) Expression level of HLA‐DMA in normal tissues and tumor tissues in TCGA. (B) Expression level of HLA‐DMA in normal tissues and paired tumor tissues in TCGA. (C‐G) Expression level of HLA‐DMA in LUAD patients with different clinical factors in TCGA [T stage (C), primary therapy outcome (D), OS event (E), age (F), and smoker (G)]. (H‐J) Expression of HLA‐DMA in tumor and unpaired para‐carcinoma tissues of the GSE10072, GSE19188 and GSE33532 datasets in the GEO database, respectively. (K‐M) HLA‐DMA expression in tumor and paired adjacent tissues in the GSE10072, GSE19188 and GSE33532 datasets, respectively.*P < 0.05,**P < 0.01, ***P < 0.001, ns indicated P > 0.05.
**Figure S6.** The HLA‐DMA expression in LUAD/paired adjacent tissues and cell lines. (A) Western blot showed the protein expression level of HLA‐DMA in different NSCLC patients. The upper was the representative immunoblot band, and the lower was the quantitative representation of the immunoblot with integrated optical density (IOD). T represents the LUAD tissue, N represents the paired adjacent tissues. (B) qRT‐PCR was used to detect HLA‐DMA expression in NSCLC patients (n = 15) and paired adjacent tissues (n = 15). (C) DAPI staining and HLA‐DMA protein detection in NSCLC patients tissue (n = 9) and paired adjacent tissues (n = 9). (D) The result of quantified staining. (E) Western blot showed the protein expression level of HLA‐DMA in cell lines. The representative immunoblot band was on the left and the lower was the quantitative representation of the immunoblot with IOD was on the right. *P < 0.05,**P < 0.01, ***P < 0.001, ns indicated P > 0.05. The scale bar = 20 μm.
**Figure S7.** KM survival curves of high‐ and low‐HLA‐DMA expression group in TCGA and GEO databases. (A) Kaplan–Meier showed the OS probability of all LUAD patients from TCGA database. (B‐E) Subgroup analysis for age over 65 years (B), T2 (C), Stage I (D), Smoker (E), and LUAD patients in the GEO datasets: GSE37745 (F), and GSE50081 (G).
**Figure S8.** Diagnostic value of HLA‐DMA expression in LUAD. (A) ROC curve analysis for HLA‐DMA. (B) The univariate Cox regression. (C) The multivariate Cox regression. (D) Nomogram survival prediction chart for predicting the 1‐, 3‐, and 5‐year overall survival rates. (E) Calibration curve for the overall survival nomogram model. The dashed diagonal line represents the ideal nomogram, and the blue line, red line and orange line represent the 1‐year, 3‐year and 5‐year of the observed nomogram.
**Table S1.** The Clinicopathological features of the LUAD patients.

**Table S2.** The function of S phase‐related genes.

## Data Availability

The datasets generated and/or analyzed during the current study are available in the TCGA repository (https://portal.gdc.cancer.gov/projects/TCGA) and GEO database (accession number: GSE10072, GSE19188, GSE33532, GSE50081, GSE37745) (https://www.ncbi.nlm.nih.gov/geo/). All original data generated in the present study can be directed to Yajie Huang (2679588968@qq.com).
