# Supplementary material for: Prognostic gene HLA‐DMA associated with cell cycle and immune infiltrates in LUAD
Source: Clin Respir J. 2023 Nov 16;17(12):1286–300. doi: 10.1111/crj.13716 (PMC10730455; doi:10.1111/crj.13716)
Supplement: Supplementary file 1 — Figure S1. Analysis workflow of this study. Figure S2. The correlation between scores and the survival/clinicopathological staging characteristics of LUAD patients. (A) Kaplan–Meier survival analysis of high‐ or low‐ score group determined by the median of Immune Score. Log‐rank P = 0.01. (B) Kaplan–Meier survival curve for Stromal Score. Log‐rank P = 0.064. (C‐F) The correlation of Immune Score with T, N, M classification and clinical stage. (G‐J) The correlation of Stromal Score with T, N, M classification and clinical stage. Figure S3. Vocalno plot, Venn plots, GO and KEGG analysis for TME‐related DEGs. (A) Based on Stromal Score comparison, 2192 genes were up‐regulated and 2680 genes down‐regulated in the high score than the low score group after propensity analysis using limma package algorithm. (B) Vocalno plot for DEGs in Immune Score, similar with (A). (C‐D) Venn plots showing common down‐regulated and up‐regulated DEGs shared by Immune Score and Stromal Score. (E) KEGG enrichment analysis for 655 TME‐related DEGs. (F‐H) Functional enrichment analysis including GO: BP, GO: CC, GO: MF, respectively. Figure S4. PPI network and univariate COX. (A) Top three modules in the PPI networks. (B) The 21 genes with node degrees≥10. (C) Univariate COX regression analysis with 655 DEGs, listing the top 30 significant factors with P value from small to large. (D) Venn plot intersected the TME‐related prognostic hub gene shared by leading 21 nodes in PPI and top 30 significant factors in COX. Figure S5. The HLA‐DMA expression in LUAD and control in TCGA and validation in GEO database. (A) Expression level of HLA‐DMA in normal tissues and tumor tissues in TCGA. (B) Expression level of HLA‐DMA in normal tissues and paired tumor tissues in TCGA. (C‐G) Expression level of HLA‐DMA in LUAD patients with different clinical factors in TCGA [T stage (C), primary therapy outcome (D), OS event (E), age (F), and smoker (G)]. (H‐J) Expression of HLA‐DMA in tumor and unpaired para‐carci [file CRJ-17-1286-s001.docx]

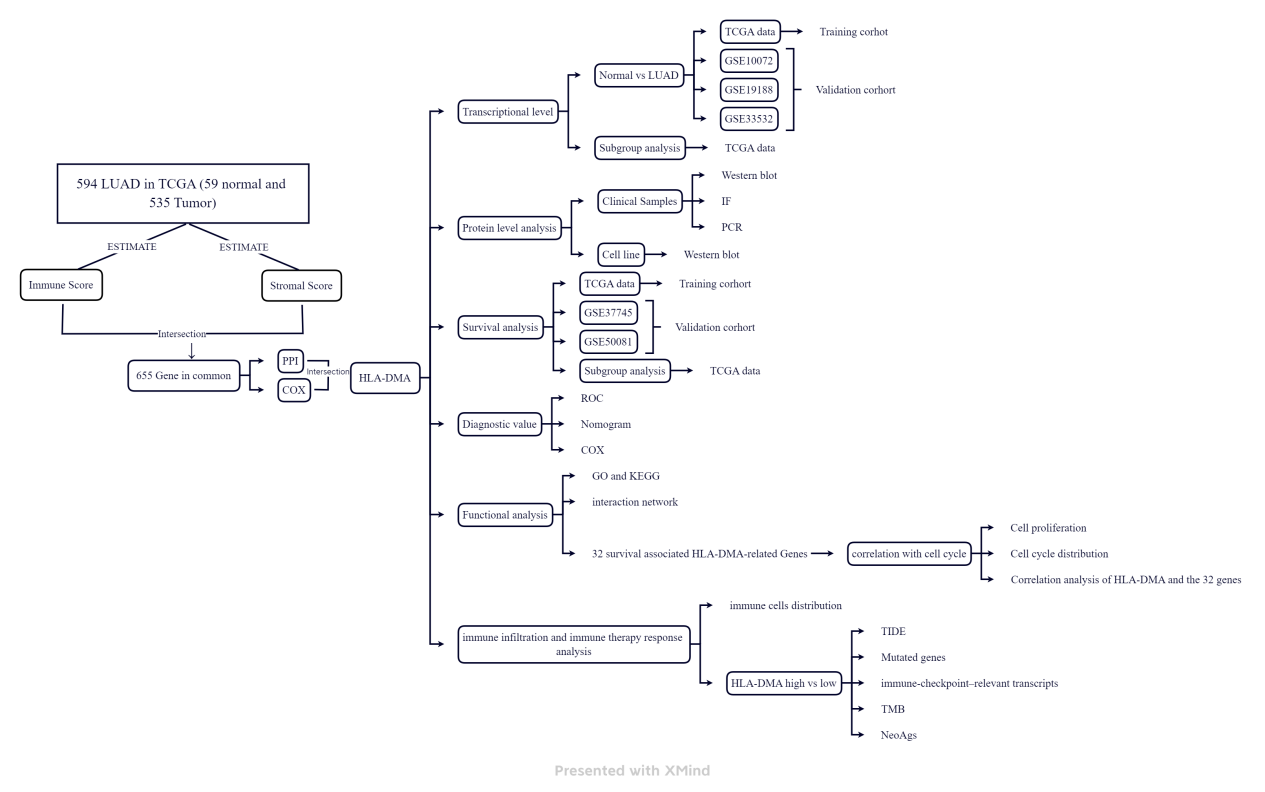


Figure S1. Analysis workflow of this study.


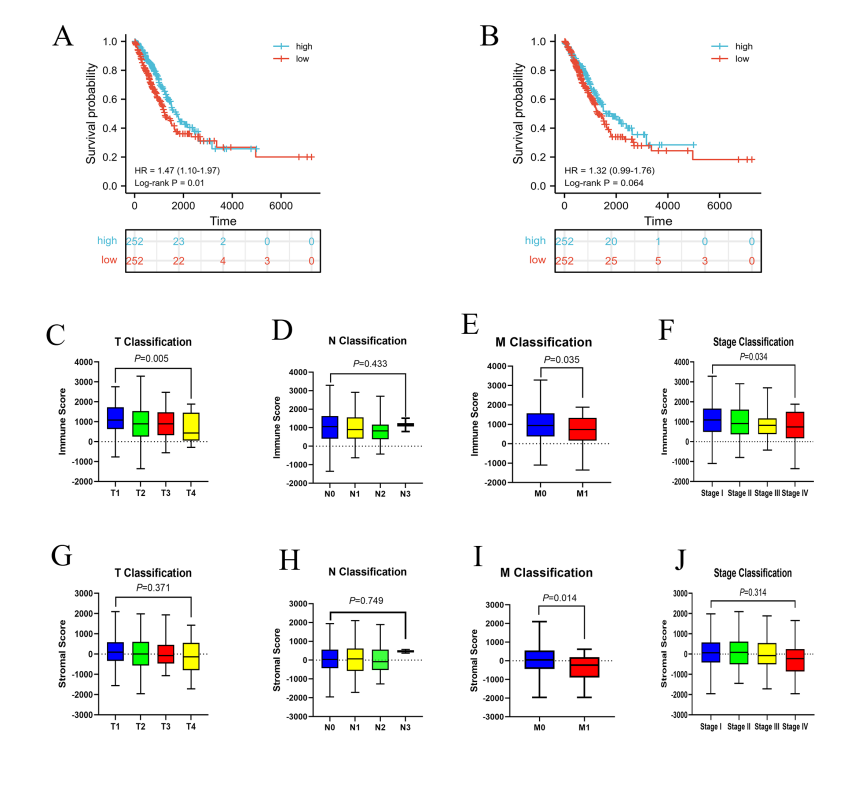


Figure S2. The correlation between scores and the survival/clinicopathological staging characteristics of LUAD patients. (A) Kaplan-Meier survival analysis of high- or low- score group determined by the median of Immune Score. Log-rank P=0.01. (B) Kaplan-Meier survival curve for Stromal Score. Log-rank P=0.064. (C-F) The correlation of Immune Score with T, N, M classification and clinical stage. (G-J) The correlation of Stromal Score with T, N, M classification and clinical stage.


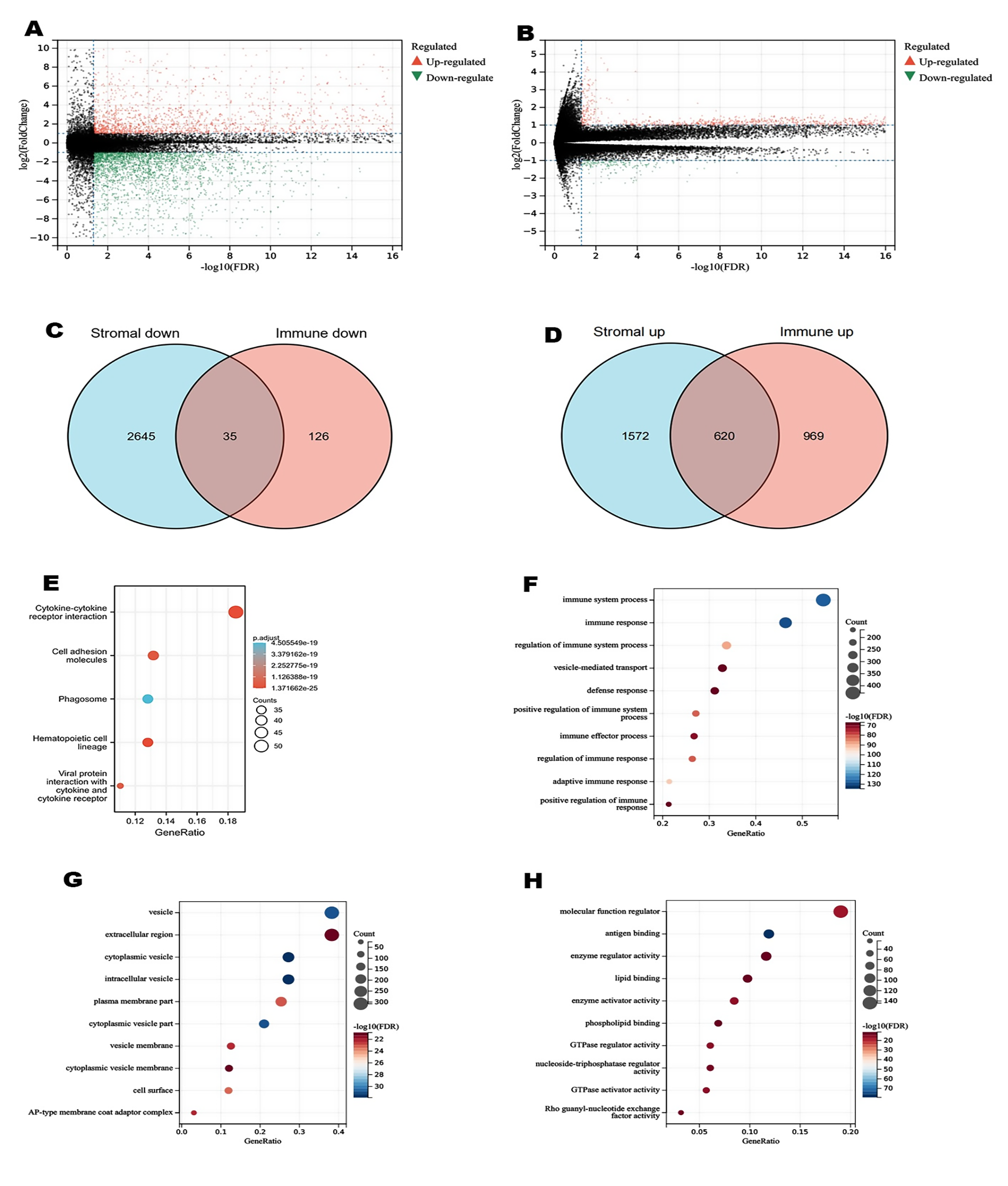


Figure S3. Vocalno plot, Venn plots, GO and KEGG analysis for TME-related DEGs. (A) Based on Stromal Score comparison, 2192 genes were up-regulated and 2680 genes down-regulated in the high score than the low score group after propensity analysis using limma package algorithm. (B) Vocalno plot for DEGs in Immune Score, similar with (A). (C-D) Venn plots showing common down-regulated and up-regulated DEGs shared by Immune Score and Stromal Score. (E) KEGG enrichment analysis for 655 TME-related DEGs. (F-H) Functional enrichment analysis including GO: BP, GO: CC, GO: MF, respectively.


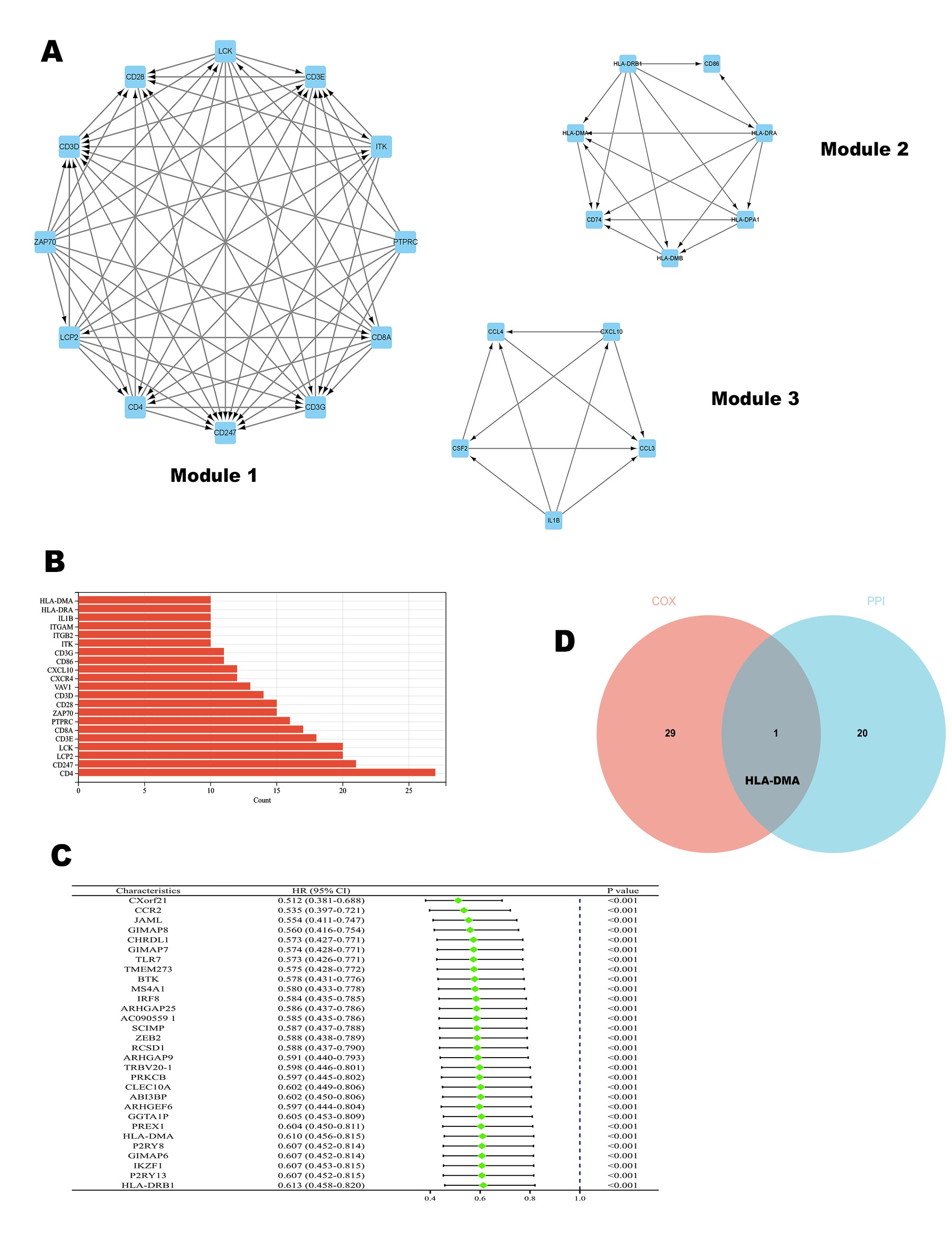


Figure S4. PPI network and univariate COX. (A) Top three modules in the PPI networks. (B) The 21 genes with node degrees≥10. (C) Univariate COX regression analysis with 655 DEGs, listing the top 30 significant factors with P value from small to large. (D) Venn plot intersected the TME-related prognostic hub gene shared by leading 21 nodes in PPI and top 30 significant factors in COX.


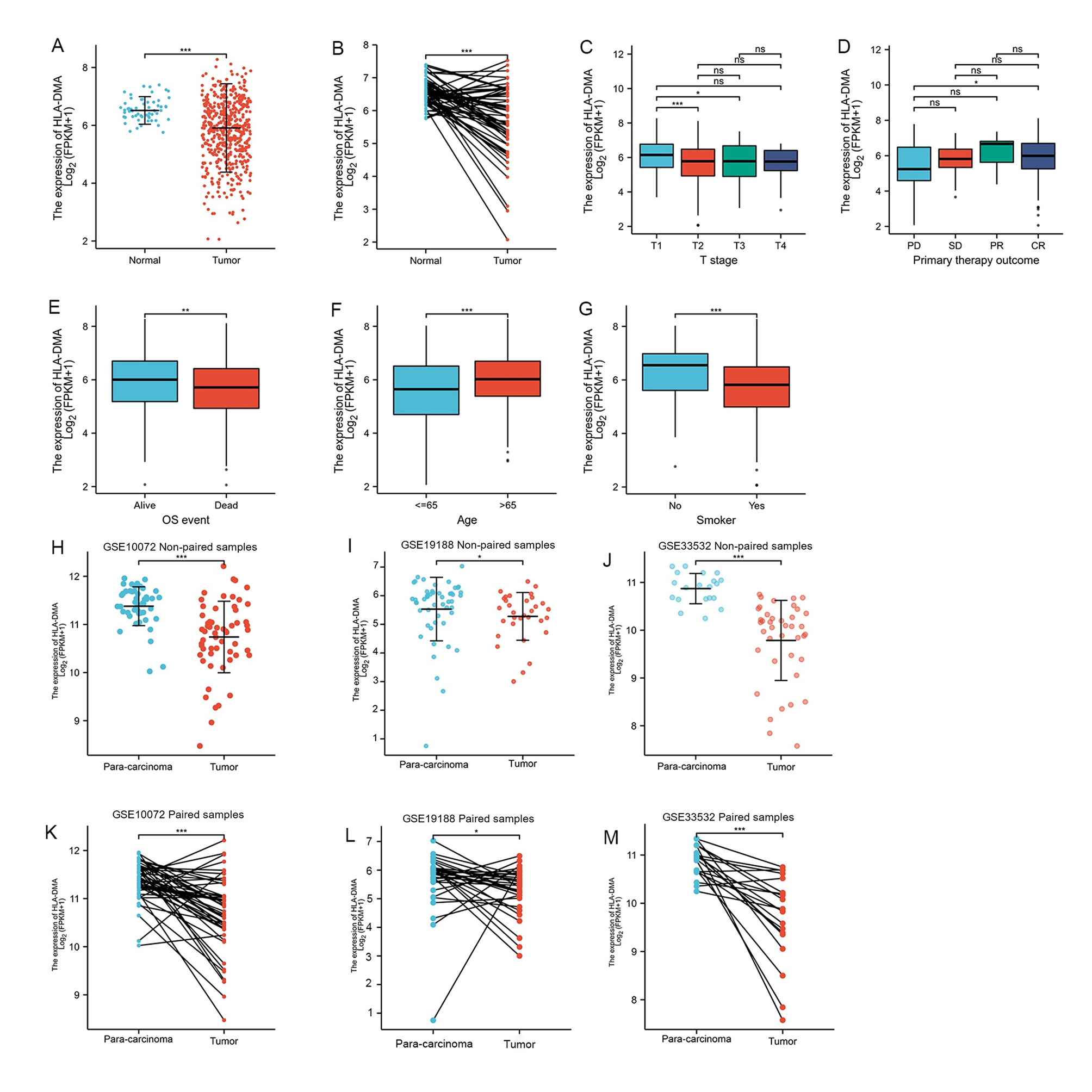


Figure S5. The HLA-DMA expression in LUAD and control in TCGA and validation in GEO database. (A) Expression level of HLA-DMA in normal tissues and tumor tissues in TCGA. (B) Expression level of HLA-DMA in normal tissues and paired tumor tissues in TCGA. (C-G) Expression level of HLA-DMA in LUAD patients with different clinical factors in TCGA [T stage (C), primary therapy outcome (D), OS event (E), age (F), and smoker (G)]. (H-J) Expression of HLA-DMA in tumor and unpaired para-carcinoma tissues of the GSE10072, GSE19188 and GSE33532 datasets in the GEO database, respectively. (K-M) HLA-DMA expression in tumor and paired adjacent tissues in the GSE10072, GSE19188 and GSE33532 datasets, respectively.*P<0.05,**P<0.01, ***P<0.001, ns indicated P>0.05.

Figure S6. The HLA-DMA expression in LUAD/paired adjacent tissues and cell lines. (A) Western blot showed the protein expression level of HLA-DMA in different NSCLC patients.The upper was the representative immunoblot band, and the lower was the quantitative representation of the immunoblot with integrated optical density (IOD). T represents the LUAD tissue, N represents the paired adjacent tissues. (B) qRT-PCR was used to detect HLA-DMA expression in NSCLC patients (n=15) and paired adjacent tissues (n=15). (C) DAPI staining and HLA-DMA protein detection in NSCLC patients tissue (n=9) and paired adjacent tissues (n=9). (D) The result of quantified staining. (E) Western blot showed the protein expression level of HLA-DMA in cell lines.The representative immunoblot band was on the left and the lower was the quantitative representation of the immunoblot with IOD was on the right. *P<0.05,**P<0.01, ***P<0.001, ns indicated P>0.05. The scale bar=20µm.


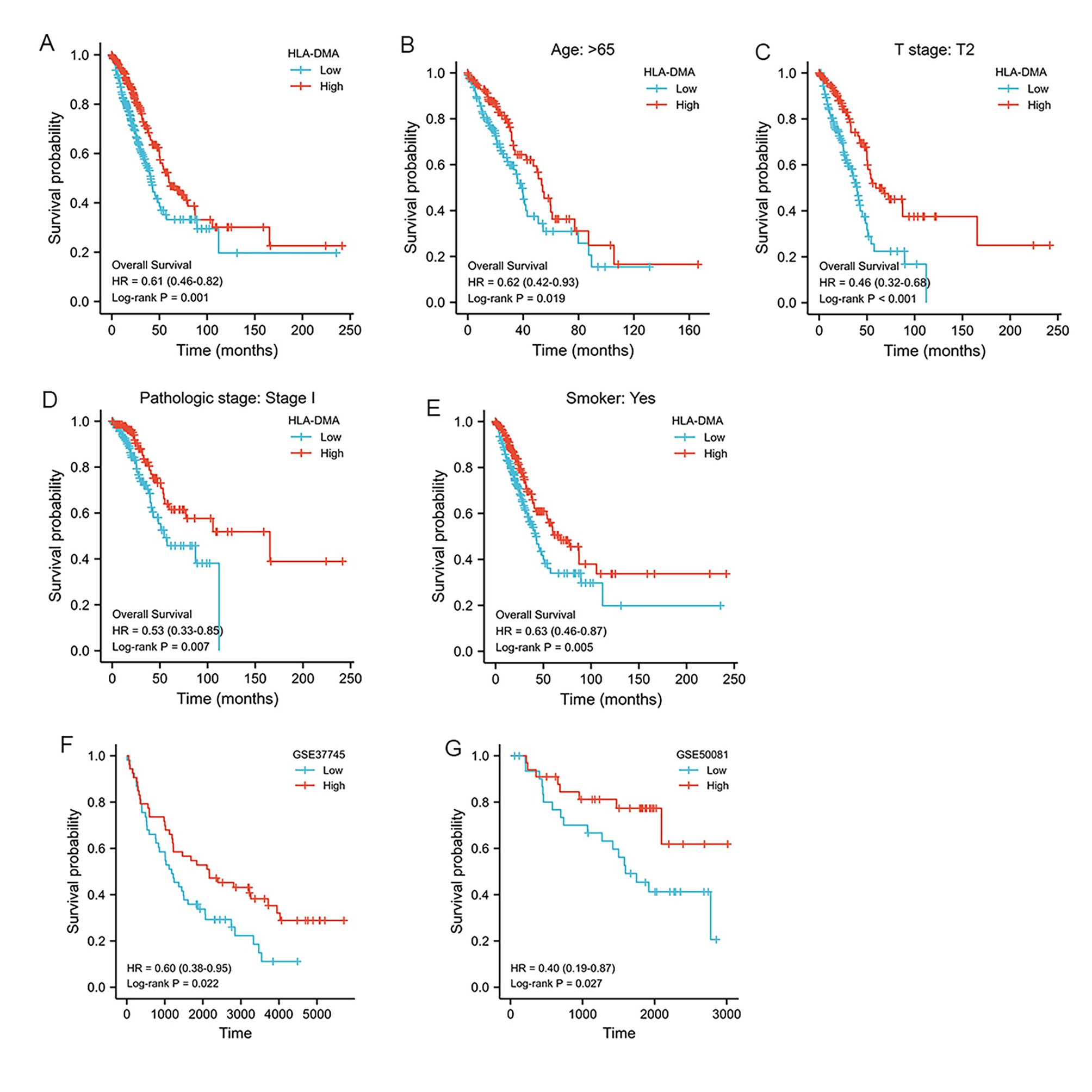


Figure S7. KM survival curves of high- and low-HLA-DMA expression group in TCGA and GEO databases. (A) Kaplan-Meier showed the OS probability of all LUAD patients from TCGA database. (B-E) Subgroup analysis for age over 65 years (B), T2 (C), Stage I (D), Smoker (E), and LUAD patients in the GEO datasets: GSE37745 (F), and GSE50081 (G).


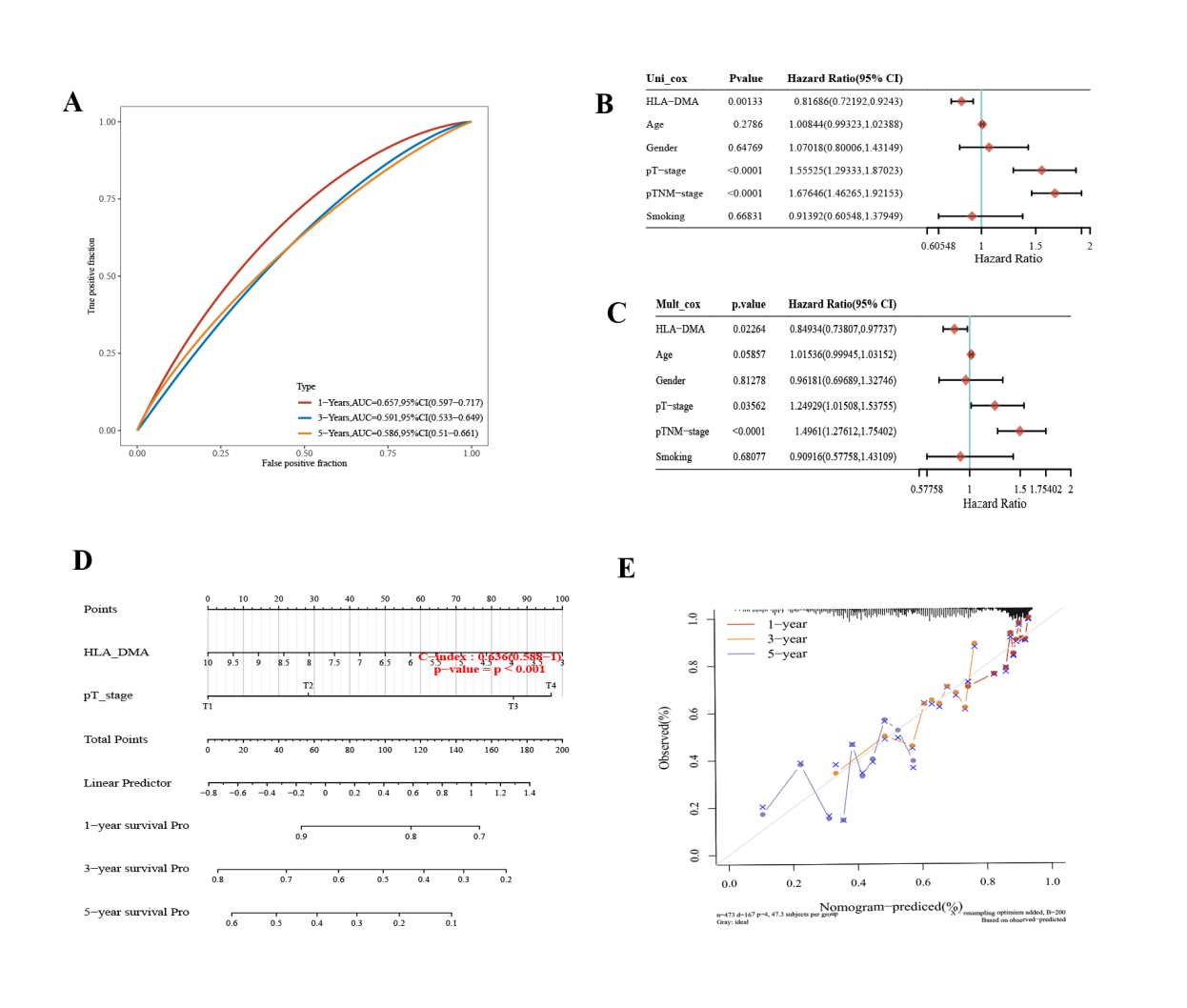


Figure S8. Diagnostic value of HLA-DMA expression in LUAD. (A) ROC curve analysis for HLA-DMA. (B) The univariate Cox regression. (C) The multivariate Cox regression. (D) Nomogram survival prediction chart for predicting the 1-, 3-, and 5-year overall survival rates. (E) Calibration curve for the overall survival nomogram model. The dashed diagonal line represents the ideal nomogram, and the blue line, red line and orange line represent the 1-year，3-year and 5-year of the observed nomogram.

Table S1. [The Clinicopathological features of the LUAD patients](https://bmccancer.biomedcentral.com/articles/10.1186/s12885-019-6311-z)

| Characteristic | Numbers |
| --- | --- |
| Age |  |
| ≥60 | 20 |
| <60 | 9 |
| Gender |  |
| Male | 21 |
| Female | 8 |
| Smoking Status |  |
| Ever | 20 |
| Never | 9 |
| Tumor Size |  |
| ≥3cm | 25 |
| <3cm | 4 |
| Clinical Stages |  |
| I-II | 25 |
| III-IV | 4 |
| Lymph node Status |  |
| N0 | 19 |
| N1-N3 | 10 |
| Metastasis |  |
| M0 | 23 |
| M1 | 6 |
