# Supplementary material for: Prognostic gene HLA‐DMA associated with cell cycle and immune infiltrates in LUAD
Source: Clin Respir J. 2023 Nov 16;17(12):1286–300. doi: 10.1111/crj.13716 (PMC10730455; doi:10.1111/crj.13716)
Supplement: Supplementary file 2 — Table S2. The function of S phase‐related genes. [file CRJ-17-1286-s002.docx]

| **Gene name** | **Gene function** |
| --- | --- |
| Cyclin C (CCNC) | G1/S specific cyclin; negative growth regulator |
| Cyclin D1 (CCND1) | G0-G1/S progression with cdk4 and cdk6 |
| Cyclin E2 (CCNE2) | G1/S progression with cdk2; histone phosphorylation. |
| P107 (RBL1) | Retinoblastoma-like 1, regulates cell cycle in S-phase |
| Cyclin-dependent kinase 2 (CDK2) | Activation of DNA replication at S phase |
| E2F transcription factor 1 (E2F1) | G1/S transition; S-phase gene |

Table S2. The function of S phase-related genes.
